# Blood and Salivary Cortisol Variations in Athletes in Relation to Cardiopulmonary Exercise Testing

**DOI:** 10.3390/medicina59101726

**Published:** 2023-09-27

**Authors:** Cezar Honceriu, Mihai Roca, Alexandru Dan Costache, Beatrice Abălașei, Lucian Popescu, Alexandru Rareș Puni, Alexandra Maștaleru, Andra Oancea, Andrei Drugescu, Cristina Adam, Ovidiu Mitu, Irina-Iuliana Costache, Maria Magdalena Leon, Iulia Cristina Roca, Veronica Mocanu, Florin Mitu

**Affiliations:** 1 Faculty of Physical Education and Sports, “Alexandru-Ioan Cuza” University, 700115 Iasi, Romania; cezar.honceriu@uaic.ro (C.H.); beatrice.abalasei@uaic.ro (B.A.); lucian_popescu2009@yahoo.com (L.P.); 2 Medical I Department, Faculty of Medicine, University of Medicine and Pharmacy “Grigore T. Popa”, 700115 Iasi, Romania; alexandra.mastaleru@gmail.com (A.M.); andra.radulescu@yahoo.com (A.O.); andreidrugescu@yahoo.com (A.D.); mituovidiu@yahoo.co.uk (O.M.); irina.costache@umfiasi.ro (I.-I.C.); leon_mariamagdalena@yahoo.com (M.M.L.); mitu.florin@yahoo.com (F.M.); 3 Department of Cardiovascular Rehabilitation, Clinical Rehabilitation Hospital, 700661 Iasi, Romania; 4 Centre of Interdisciplinary Research in Human Motricity Science, “Alexandru-Ioan Cuza” University, 700115 Iasi, Romania; punirares@yahoo.com; 5 Department of Cardiology, “St. Spiridon” Emergency County Hospital, 700111 Iasi, Romania; 6 “St. Spiridon” Emergency County Hospital, 700111 Iasi, Romania; iuliaroca@yahoo.com; 7 Surgery II Department, Faculty of Medicine, University of Medicine and Pharmacy “Grigore T. Popa”, 700115 Iasi, Romania; 8 Morpho-Functional Sciences II Department, Faculty of Medicine, University of Medicine and Pharmacy “Grigore T. Popa”, 700115 Iasi, Romania; veronica.mocanu@gmail.com; 9 Romanian Academy of Medical Sciences, 927180 Bucharest, Romania; 10 Romanian Academy of Scientists, 050044 Bucharest, Romania

**Keywords:** football players, cortisol, cardiopulmonary exercise testing

## Abstract

*Background and Objectives*: Cortisol is a valuable marker for assessing the body’s response to any form of stress. We conducted this study in order to evaluate the variations of salivary and serum cortisol levels in professional football players in relation to cardiopulmonary exercise testing (CPET) and their significance in potentially evaluating overtraining in athletes. Also, the question of whether salivary cortisol determination could fully substitute serum sampling was addressed. *Materials and Methods*: A total of 19 male professional football players were evaluated by measuring serum cortisol levels at rest (T0) and immediately after a CPET (T1) and salivary cortisol levels at rest (T0), 10 min after a CPET (T2), and 30 min after a CPET (T3). *Results*: T0 serum cortisol showed a statistically significant correlation with the oxygen uptake at the anaerobic threshold divided by the body weight (VO_2_-AT/weight), as did the T2 salivary cortisol with the maximum oxygen uptake at the anaerobic threshold (VO_2_-AT) and VO_2_-AT/weight. T0 salivary cortisol was significantly correlated with the subjects’ height and the predicted O_2_ pulse. *Conclusions*: While some correlations were discovered, they are insufficient to recommend cortisol as a routine biomarker in athletes’ evaluation. However, significant correlations were established between salivary and serum determinations, meaning that the non-invasive procedure could substitute venous blood sampling.

## 1. Introduction

Professional football is often regarded as a strenuous physical activity, considering the intensity and duration of football matches (a minimum of 90 min), but also the training regimens involved, composed of both aerobic (endurance-oriented) and anaerobic (strength-oriented) exercises. Therefore, most footballers reach their peak physical performance in their early twenties and start considering retiring when they reach the age of 30. Thus, it is crucial to adapt all training sessions to the overall fitness level of the athlete. Overtraining leads to exhaustion and to a decrease in physical performance. In more severe cases, events such as muscle or joint injuries, as well as fatal situations like sudden cardiac death, may occur. Considering this, several protocols have been used over the years to better assess the body’s overall condition when subjected to such intense workouts [1,2,3].

Considering the most severe scenario, which would be sudden cardiac death in young asymptomatic athletes, several guidelines have been issued, including the 2020 European Society of Cardiology (ESC) Guidelines on sports cardiology and exercise in patients with cardiovascular disease, which tackles the evaluation of these individuals. However, even the guideline acknowledges the gaps in evidence in athlete evaluation and the need for further studies [4].

Most evaluation protocols include, as standard, a clinical examination and, optionally, a 12-lead resting electrocardiogram (ECG) and trans-thoracic echocardiography (TTE). At the moment, the stress test is not routinely recommended. However, its utility is being recognized and used on a broader scale, especially in assessing overall physical ability, if not for showing ischemic patterns or symptoms [4].

However, all these techniques may lack sensibility in highlighting undiagnosed diseases. As a consequence, there is a need to study other complementary methods that would aid in the diagnostic process together with the previously mentioned methods [5,6,7,8,9,10].

More recently, biomarkers have been included in the protocols for evaluating athletes, with frequent mentions of testosterone and cortisol. They accurately predict the imbalance between anabolic and catabolic metabolism, offering a very good indication of overtraining. They can easily be measured from blood samples, but more recently from saliva, offering a more comfortable technique for both physicians and subjects, with the possibility of sampling even in more challenging environments outside of the hospital [5,6,7,8,9,10].

Cortisol is a reliable endocrine parameter of body stress. There are many situations that induce increased (hypercortisolism/Cushing syndrome) or decreased values (hypocortisolism). However, physical effort can also have an influence without a coexistent pathology, and variations can occur in healthy individuals when subjected to intense physical stress [11,12,13,14,15].

Generally, in all individuals, there is a normal variation in the diurnal cortisol levels from the morning until the evening, which is called the diurnal cortisol slope. Considering this graphical representation, several studies have analyzed different patterns and correlated them with healthy or unhealthy behavior. As a general rule, it is normal for this slope to have a higher angle or variation during the day, while flatter patterns have been associated with poorer health outcomes. A flatter diurnal cortisol slope has been encountered in individuals with known diseases such as cancer, obesity, atherosclerosis, depression, or chronic fatigue. Inflammatory or immune system dysregulations were very strongly correlated with a flatter diurnal cortisol slope. Interestingly, cardiovascular diseases or anxiety were not strongly associated with a flatter diurnal cortisol slope in various publications. It is, however, unclear whether this pattern is diagnostic of an emerging disease or a result of an already long-ongoing process and how it may be applied in routine practice [16].

In athletes, it is useful in assessing periods of stress or overtraining, while long-term increased or decreased levels are linked to altered physical performance [17]. Over the years, several studies have highlighted different uses of cortisol determinations with regard to physical effort in athletes. However, the results are varying. One interesting study by Duclos et al. followed the relationship between cortisol and growth hormone levels post-exercise and highlighted their importance in athlete doping using products based on the two [17]. One common ground in all of them was the link between serum and salivary cortisol variations [18,19].

One important study was conducted by Mishica et al. in 2021 on athletes, which investigated the relationship between training programs, sleep duration, heart rate variability, and salivary cortisol levels. The investigation reached the following main conclusions: During test weeks, there was a substantial correlation between the lowest heart rate variability and the highest salivary cortisol levels. The decrease in heart rate variability at the middle of the training period was correlated with a decrease in the amount of hard training and training load, indicating that this reduction was not brought on by the stress of training but rather by the onset of the competition period, which was associated with an increase in muscle fatigue and stress. The decrease in sleep length was inversely correlated with both hard training periods and salivary cortisol levels when the athletes were engaged in training, suggesting that less sleep may be linked to increased weekly strain. Finally, salivary cortisol levels showed a reduction in the volume of hard training despite an increase in the quantity of easy training and training load, with a favorable correlation between changes in intense training periods and cortisol during weeks 3–5. Thus, when the volume of hard training is decreased, young endurance athletes seem to tolerate higher volumes and periods of easy training [20].

As early as 2002, Neary et al. showed a strong correlation between salivary and serum cortisol levels during the recovery period following a training session of eight physical education students (five males and three females). They postulated that either one of them could be used, with an emphasis on salivary cortisol determination, due to the non-invasive nature of its determination [18].

In 2008, Hellhammer et al. addressed the use of salivary cortisol measurements. They stated that other biomarkers are not that well correlated since some are influenced by a complex link of factors. Therefore, they proceeded to highlight the potential dissociations between salivary cortisol levels, corticotrophin-releasing factor (CRF), arginine vasopressin (AVP), adrenocorticotropic hormone (ACTH), and serum and urinary cortisol levels [19].

It was shown that cortisol levels increase with the duration and intensity of training, yet no reduction in their levels has been noted when physical activity was decreased afterward [18]. Only between peaks of maximal activity did cortisol levels regress [21].

While maximal testing is ideal in evaluating physical capabilities and performance, it does not replicate day-to-day training as well as sub-maximal testing, which offers a more physiological approach. Ideally, both types of tests should be used interchangeably [22,23,24].

Cardiopulmonary exercise testing (CPET) is a valuable procedure that allows the evaluation of multiple respiratory and cardiac parameters [25]. It can also be used in athletic performance assessment, by linking maximal oxygen uptake (VO_2_ max) to the exercise capacity and simultaneously offering a reduced risk of cardiovascular disease [26,27,28,29]. While normal individuals reach vales of 30–45 mL/kg/min, elite aerobic athletes can go as high as 80 mL/kg/min or beyond [30]. Of particular use in this category is to differentiate between athletic physiological left ventricle hypertrophy (LVH) and hypertrophic cardiomyopathy [31,32].

For some studies concerning the evaluation of athletes, gaps in the evidence have been acknowledged, either regarding the sample size or the methodology itself. While our sample size was also reduced, we used a study protocol that encompassed both serum and salivary determinations, as well as multiple measurements at different time points. The objectives were to measure the variations of both serum and salivary cortisol in relation to an induced physical stress, to establish any possible correlations and their possible use in future screening protocols, and, finally, to determine whether salivary cortisol determinations could fully substitute serum cortisol sampling, given its non-invasive nature and ease of determination.

## 2. Materials and Methods

### 2.1. Experimental Approach

We have conducted a complex cardiometabolic evaluation of professional football players, with an emphasis on cardiopulmonary exercise testing (CPET) and the blood and salivary levels of cortisol in order to evaluate the stress response of the body during strenuous activity. The study was designed following the recommendations for clinical research contained in the Helsinki Declaration of the World Medical Association, and the protocol was approved by both the Ethics Committee of the Clinical Rehabilitation Hospital in Iași, Romania, approved on 24 March 2021 and the Ethics Research Committee of the “Grigore T. Popa” University of Medicine and Pharmacy in Iași, Romania, nr. 72 approved on 25 April 2021.

### 2.2. Participants and Protocol

We evaluated a total of 19 professional football players. From the total rosters of football players from the Iași and Vaslui counties’ football centers, we selected a number of 30 players aged between 18 and 20 years. We excluded players who were injured at the time or in the recovery period post-injury, as well as those who declined participation.

All subjects were male and aged between 18 and 20 years old (mean 18.47 ± 0.841). In order to avoid any interference with the results of the test, we asked them to halt all intense training or games a minimum of 24 h before the procedures. Also, regarding the saliva samples, they were required not to ingest any foods in the morning and to avoid brushing their teeth or using any products such as mouthwash. They were admitted in the Cardiology Clinic of the Rehabilitation Hospital through day hospitalization in August and September 2021. All informed consent forms concerning the procedures and their inclusion in the study were completed prior to any examination.

### 2.3. Initial Evaluation

Upon admission, they underwent fast COVID-19 antigen testing, as this was during the pandemic period. Only if the results were negative could they undergo the rest of the evaluation. At rest, the usual blood samples were taken (e.g., full blood count, biochemistry). At this point, blood samples were also taken for measuring the resting serum cortisol levels (T0). Also, using special saliva containers, saliva samples were taken for measuring the resting salivary cortisol levels (T0).

Following the sampling of both saliva and blood, they underwent a clinical examination with the focus on the cardiovascular system (e.g., heart auscultation). Additionally, resting blood pressure (BP) and heart rate (HR) were measured.

### 2.4. Resting ECG

All subjects underwent a resting 12-lead resting ECG. Given that we were examining healthy asymptomatic individuals and considering the morphological and functional adaptation of the cardiovascular system to chronic physical stress in elite athletes, all identified patterns were analyzed considering normal, borderline, and pathological changes in athletes [33,34].

### 2.5. Cardiac Ultrasound

Standard transthoracic echocardiographic evaluation prior to the CPET assessed the cardiac function and excluded any possible contraindications. All subjects were evaluated with the M-mode, pulse wave Doppler (PWD), continuous wave Doppler (CWD), and color Doppler methods.

### 2.6. Cardiopulmonary Exercise Testing

Cardiopulmonary exercise testing (CPET), using the BTL CardioPoint software (version 2.32) and BTL-compatible equipment, was used to evaluate the functional capacity. On the cycle ergometer, we employed a progressive maximal symptom-limited CPET protocol that was specially designed for the athletes: they began at a workload of 15 Watts that was scheduled to increase by 12.5 Watts every 30 s. The testing lasted 10 to 12 min, and a 10 min rest period followed.

The most crucial CPET parameters included the following: maximum work rate (absolute value, WR (Watt), and percentage of the predicted value, WR% (%)); oxygen uptake with maximal aerobic capacity (absolute value, VO_2_ max (milliliters per minute), and percentage of the predicted value, VO_2_ max%); carbon dioxide output (VCO_2_ (milliliters per minute)); and oxygen uptake at the anaerobic threshold (AT) (milliliters per minute). As values normalized by body weight (milliliters per minute per kilogram), VO_2_, VCO_2_, and AT were also expressed.

The respiratory exchange ratio (RER) is essential in CPET interpretation. It shows if the procedures were conclusive to interpret and whether all recordings in all subjects were correctly conducted and uniform. Also, the oxygen pulse (O_2_ pulse—mL/beat) is an indirect indicator of the maximum stroke volume during effort.

The ratio of the rise in VO_2_ to the rate of increase in work rate (VO_2_/WR slope) was used to calculate metabolic efficiency. The VO_2_/WR ratio reflects the metabolic conversion of chemical energy to mechanical work during exercise. A low ratio shows impaired O_2_ delivery to the muscle [35].

Using the auscultatory approach, blood pressure was checked every two minutes, while a 12-lead ECG was captured in real time.

A subjective assessment of the severity of perceived exhaustion was made using a 6–20 Borg scale of perceived exertion in order to clinically determine the exercise’s intensity.

Following the latest recommendations, the test would be halted at the request of the patient, upon symptoms or fatigue occurrence, when the blood pressure (BP) measurement would exceed 220 mmHg for the systolic value or 120 mmHg for the diastolic value, or when suggestive ischemic ECG patterns would appear on the dynamic recording.

### 2.7. Cortisol Determinations

Peripheral venous blood was collected at rest for the basic laboratory parameters and also to determine the serum cortisol values at rest (T0). At the same time, saliva collection kits were used to collect the required specimen to determine the salivary cortisol level at rest (T0). Immediately after finalizing the CPET, another sample was taken to determine the level of cortisol reached in the blood post-effort (T1). All blood sampling was obtained from the antecubital veins and in dedicated vacutainers. Ten minutes after finishing the CPET (T2) and thirty min, respectively (T3), saliva collecting kits were used to sample further specimens to determine salivary cortisol levels at three different time points in total (T0, T2, and T3).

The determinations were accomplished through the ELISA procedure, using the semi-automated ELISA, model UT6500, produced by MRC, with DiaMetra kits.

### 2.8. Data Analysis and Statistics

SPSS 20.0 (Statistical Package for the Social Sciences, Chicago, IL, USA) was used to analyze the data. For continuous variables, the data were shown as mean and standard deviation (SD), or as median and interquartile range. Due to the small number of subjects involved in the study, the distribution of these variables did not meet the assumption of normality. Hence, they were compared using the non-parametric Mann–Whitney U test. The Spearman correlation coefficients were calculated to evaluate correlations between continuous variables for the same purpose. A two-sided *p*-value of 0.05 or lower was regarded as statistically significant. Bonferroni correction was applied as a multiple-comparison correction method.

## 3. Results

The anthropometric data were uniformly distributed. Fifteen subjects had a normal BMI (18.55–24.99 kg/m^2^), while four were overweight (BMI 25 kg/m^2^ or above), yet none had an abdominal circumference above 102 cm.

The resting SBP, DBP, and HR were within normal ranges, and no significant pathological encounters were noted during the clinical examination. Consequently, the resting 12-lead ECG and TTE showed no pathological aspects that would contraindicate the CPET.

All the participants reached the anaerobic threshold during the CPET with a respiratory exchange ratio (RER) above 1.00 recorded. In 12 cases, the CPET was halted due to SBP values of 220 mmHg or above, while in the others, it was halted due to muscle fatigue. No ischemic symptoms of ECG patterns were recorded (see Table 1).

Serum cortisol dynamics presented a decrease without statistical significance from T0 to T1 (*p* = 0.421). Saliva cortisol presented a decrease without statistical significance from T0 to T2 (*p* = 0.409) and on the edge of statistical significance from T0 to T3 (*p* = 0.055) (see Table 2).

All recorded CPET parameters were subjected to statistical analysis, especially with both serum and salivary cortisol determinations (see Table 3 and Table 4).

**Figure 1:**
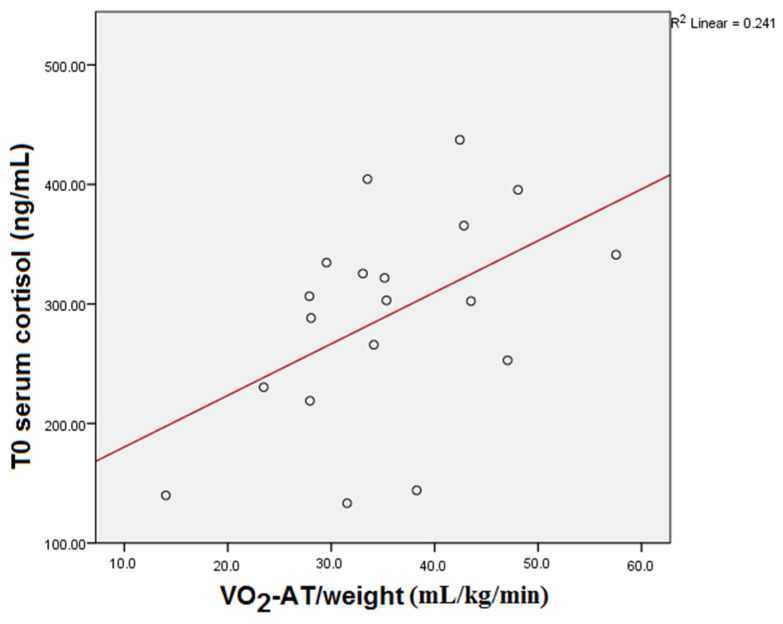
T0 serum cortisol and VO_2_-AT/weight correlation.

T0 serum cortisol showed a significant statistical correlation with the VO_2_-AT/weight reached by the subjects during CPET (*p* = 0.048) (see Figure 1). However, statistical significance is altered if the Bonferroni correction is applied (*p* = 0.096).

**Figure 2:**
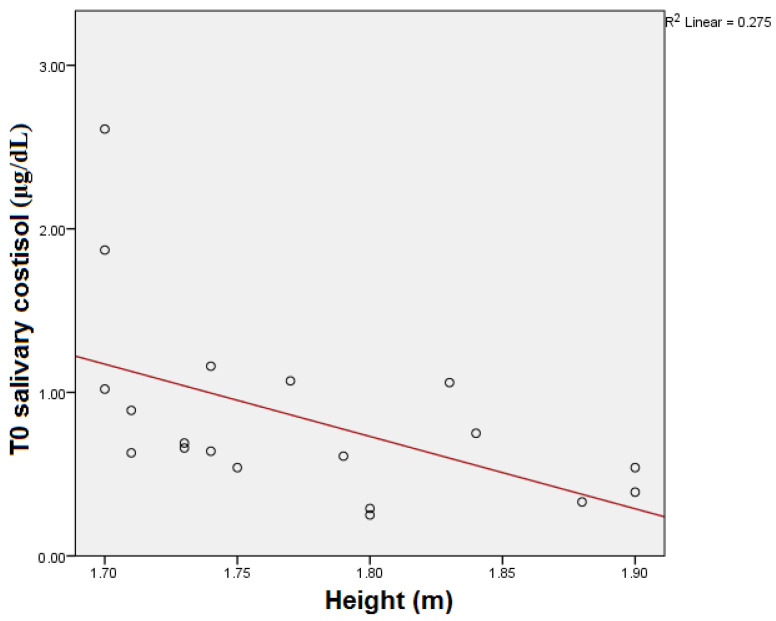
T0 salivary cortisol and height correlation.

T0 salivary cortisol showed significant statistical correlation with the height of the subjects (*p* = 0.005) (see Figure 2). Statistical significance was also maintained after the Bonferroni correction (*p* = 0.015).

**Figure 3:**
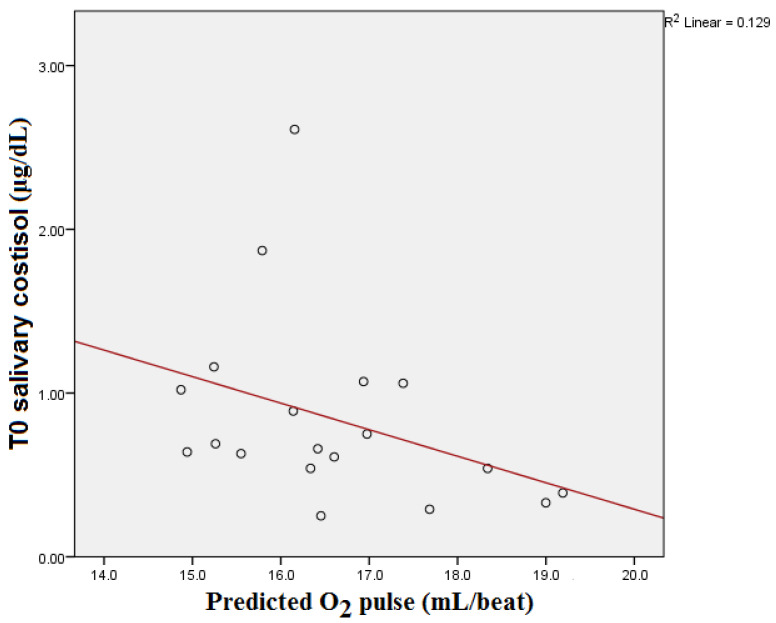
T0 salivary cortisol and predicted O_2_ pulse correlation.

T0 salivary cortisol showed a significant statistical correlation with the predicted O_2_ pulse of the subjects (*p* = 0.032) (see Figure 3). However, statistical significance was lost after the Bonferroni correction (*p* = 0.096).

**Figure 4:**
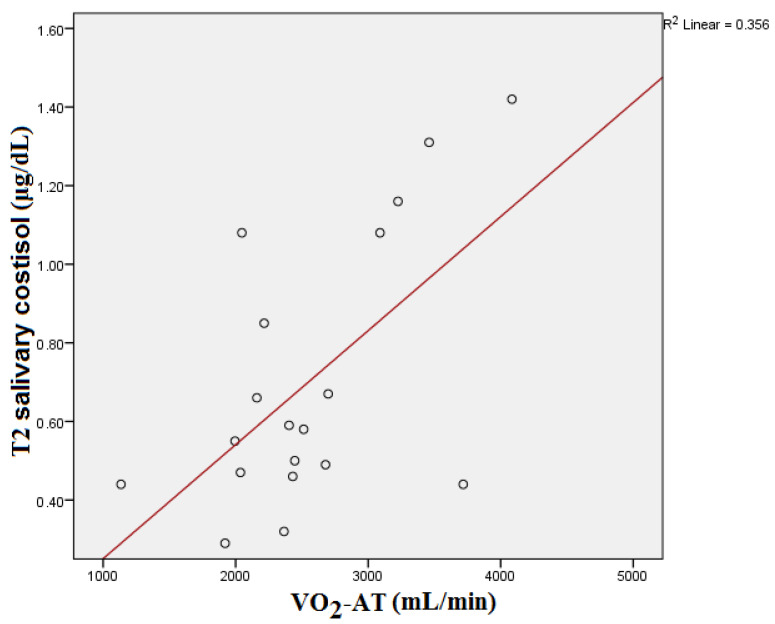
T2 salivary cortisol and VO_2_-AT correlation.

T2 salivary cortisol showed a significant statistical correlation with the VO_2_-AT reached by the subjects during CPET (*p* = 0.029) (see Figure 4). After the Bonferroni correction, the statistical significance was also altered (*p* = 0.087).

**Figure 5:**
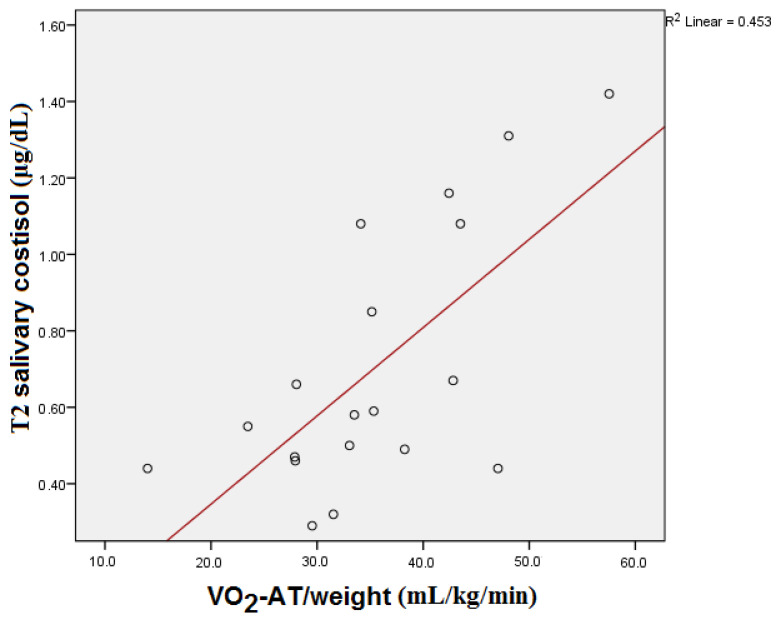
T2 salivary cortisol and VO_2_-AT/weight correlation.

T2 salivary cortisol showed significant statistical correlation with the VO_2_-AT/weight reached by the subjects during CPET (*p* = 0.005) (see Figure 5). Statistical significance was also maintained after the Bonferroni correction (*p* = 0.015).

Another comparison was drawn between the serum and salivary cortisol variations in order to observe any possible correlations between the two types of determinations (see Table 5). Given the multiple *p*-values below 0.05, significant correlations were established between both types of determinations, meaning that the non-invasive procedure could substitute the blood sampling, given its many advantages.

## 4. Discussion

Biomarkers are gaining more and more use in present-day sports medicine. While serum measurements are the mainstay, saliva sampling is increasing due to its ease of collecting as a technique also in an out-of-hospital setting. However, the issue that needs addressing is whether saliva cortisol measurements are statistically correlated to serum determinations and if they can completely replace venous blood collection [36,37].

This has been debated by different studies, which reached conflicting conclusions. While some studies found some correlations between serum and salivary determinations, either for cortisol, testosterone, or other markers of overtraining, others concluded that there are significant differences between serum and salivary sampling that do not replace the determination of one with the other [9].

Obmiński and Stupnicki found strong correlations between the salivary and serum determination of the testosterone to cortisol ratio in a population of triathletes and karate athletes [38]. Such correlations were also noted between baseline serum and salivary cortisol levels in football players and for testosterone values in athletes [39,40].

Interestingly, Cadore et al. found significant correlations between baseline serum and salivary cortisol determinations yet not between serum and salivary testosterone, a finding supported by Hayes et al. [9,41].

Our study is comparable in its design and sample size to others that have investigated cortisol variations in athletes and its possible use.

Mishika et al. studied the variations of salivary cortisol in eight young endurance athletes over a period of 7 weeks in relation to their heart rate variability (HRV), sleep duration, and response to training. They highlighted the importance of sleep during the training season and the negative correlation of salivary cortisol with nocturnal HRV. The recovery time is also longer because the HRV is lower during rest periods compared to competitive seasons. Again, cortisol values and training duration did not show any significant statistical difference [20].

Salivary cortisol is considered a reliable marker of stress with a higher use, especially due to its non-invasive nature of obtaining samples. However, many studies have shown that it actually increases during high-intensity exercise bouts and does not offer a significant correlation during low-intensity and moderate-intensity training [19,20,42].

Furthermore, as Hynynen et al. have concluded in their study, overtraining in athletes does not exhibit significant differences compared to control groups, while Feitosa et al. maintain that genetics and individual predispositions should also be taken into consideration for baseline and cortisol level variations [43,44].

Several studies have also shown that there is also no significant difference between long-duration, low-intensity training and high-intensity, short-interval bouts in cortisol levels. Yet, a difference was noted when comparing training periods to competition season, when cortisol levels were significantly higher, as suggested by the added psychological stress factor [45,46].

Apart from just physical activity and strain, lifestyle is essential for athletes, and sleep quality and duration have been confirmed to correlate with physical performance. This is also highlighted by higher cortisol levels in sleep-depraved athletes who also showed reduced training results and increased recovery times [47,48].

Several other contributing factors to cortisol variations are the parasympathetic form of overtraining syndrome in athletes, which explains the delayed fatigue that occurs during prolonged periods of training or competition, as well as the associated muscle damage and anti-inflammatory processes. These are accompanied by increases in cortisol levels [49,50,51].

Yet, given the many advantages it offers, the sole use of salivary cortisol determination is a valuable tool in athletes’ assessment, made even more accessible through the use of commercially available assays [52].

Of particular importance from the established correlations are the ones between serum and salivary cortisol at different time points and the oxygen uptake at the anaerobic threshold. This highlights how the change in metabolism from aerobic to anaerobic is associated with increased cortisol levels during intense bouts of physical exercise. The use of CPET to induce a controlled physical effort to further examine the variations of cortisol levels is a novelty, as very few studies have used a controlled form of physical stress [53,54].

While trying to highlight the strengths of our study, we would like to point out the homogenous sample, composed of all males, participating in the same type of physical activity (professional football players) and having a narrow age interval (18 to 21 years of age), relevant to similar peak physical performance when subjected to an intense controlled workout. Additionally, we measured both serum and salivary cortisol instead of relying on only one type of determination. This allowed for a comparison of both types of sampling techniques to assess whether one can substitute the other and if only one type would suffice for future studies.

Concerning the limitations of our study, we would like to acknowledge the small sample size, comprising only 19 subjects. While comparable to other studies performed on athletes, the sample did not allow for more complex statistical analysis or stronger correlations to be obtained. Also, the methodology would have required a more frequent number of measurements of both serum and salivary cortisol in order to observe a better dynamic.

The conclusions of the study are bidirectional. Firstly, while some correlations were established using non-parametric tests and fewer were confirmed following the Bonferroni analyses, these are insufficient and unclear regarding the implementation and interpretation of the screening of athletes. Secondly, the correlations between serum and salivary cortisol could indicate that in the future, the non-invasive determination (salivary sampling) could be used instead of the other, given its more accessible technique and higher comfort for the patient. However, in order to ascertain these findings, further studies are required, preferably on larger samples and with a higher number of measurements.

## 5. Conclusions

Both serum and salivary cortisol values showed some correlations with anthropometric parameters and those recorded during CPET, especially during the transition from aerobic to anaerobic metabolism. These correlations cannot be, however, substantiated at the moment and do not show the use of cortisol determination in athletes’ evaluation. However, significant correlations were established between salivary and serum determinations, meaning that the non-invasive procedure could substitute venous blood sampling. Further studies on larger samples are needed.

## Figures and Tables

**Table 1:** CPET parameters.

Parameter	Median	Interquartile Range
VO_2_ max (mL/min)	3753	795
VO_2_ max body weight (mL/min/kg)	50.06	11.1
%VO_2_ max	109.00	25.00
VO_2_@AT (mL/min)	2431.00	1042.00
VO_2_@AT body weight (mL/min/kg)	34.11	14.77
RER	1.11	0.11
VE/VCO_2_	22.35	5.35
ΔVO_2_/ΔWR (mL/min/Watt)	13.2	2.34
WR max (Watt)	248.00	37
%WR max	86.00	7.00
O_2_ pulse (mL/beat)	21.7	5.2
%O_2_ pulse	134.32	29.71
HR max (bpm)	167	16
%HR max	83	8
HR_rez (bpm)	107	14
SBP max (mmHg)	220.0	30.0
DBP max (mmHg)	85.0	5.0

Abbreviations: VO_2_ max—maximum oxygen uptake; VO_2_ max body weight—ratio of maximum oxygen uptake to body weight; %VO_2_ max—percentage of maximum oxygen uptake from the predicted value; VO_2_@AT—oxygen uptake at the anaerobic threshold; VO_2_@AT body weight—ratio of oxygen uptake at the anaerobic threshold to body weight; RER—respiratory exchange ratio; VE/VCO_2_—ventilatory equivalent for carbon dioxide; ΔVO_2_/ΔWR—slope of the relation VO_2_–power in W; WR max—maximum load; %WR max—percentage of maximum load from the predicted value; O_2_ pulse—oxygen pulse; %O_2_ pulse—percentage of oxygen pulse from the predicted value; HR max—maximum heart rate; %HR max—percentage of maximum heart rate from the predicted value; HR_rez—heart rate reserve; SBP max—maximum systolic blood pressure; DBP max—maximum diastolic blood pressure.

**Table 2:** Cortisol determinations.

Parameter	Median	Interquartile Range
T0 serum cortisol (ng/mL)	303.01	110.83
T1 serum cortisol (ng/mL)	279.92	207.52
T0 saliva cortisol (ug/dL)	0.66	0.52
T2 saliva cortisol (ug/dL)	0.58	0.62
T3 saliva cortisol (ug/dL)	0.55	0.22

**Table 3:** Serum cortisol and CPET parameters correlations.

	T0 Serum Cortisol (ng/mL)		T1 Serum Cortisol (ng/mL)	
	r	*p*	r	*p*
VO_2_ max (mL/min)	0.009	0.972	−0.251	0.300
VO_2_ max/weight (mL/kg/min)	0.151	0.538	−0.163	0.505
VO_2_ max % predicted	0.087	0.723	−0.237	0.328
VO_2_-AT (mL/min)	0.416	0.077	0.312	0.193
VO_2_-AT/weight (mL/kg/min)	0.460	0.048	0.372	0.117
RER	−0.063	0.799	−0.209	0.391
VE/VCO_2_	−0.179	0.464	0.168	0.491
ΔVO_2_/ΔWR (mL/min/Watt)	−0.028	0.909	−0.289	0.229
WR (Watt)	0.101	0.679	−0.230	0.344
WR % predicted	0.156	0.524	−0.333	0.164
Predicted O_2_ pulse	−0.340	0.154	−0.102	0.679
O_2_ pulse (mL/beat)	−0.024	0.923	−0.281	0.244
O_2_ pulse % predicted	0.082	0.737	−0.249	0.304
HR max (bpm)	0.163	0.504	0.076	0.756
HR max % predicted	0.177	0.468	0.079	0.747
SBP max (mmHg)	−0.026	0.916	0.351	0.141
DBP max (mmHg)	−0.288	0.231	0.018	0.942

**Table 4:** Salivary cortisol and CPET parameters correlations.

	T0 Salivary Cortisol (µg/dL)		T2 Salivary Cortisol (µg/dL)		T3 Salivary Cortisol (µg/dL)	
	r	*p*	r	*p*	r	*p*
VO_2_ max (mL/min)	0.017	0.946	−0.161	0.511	−0.178	0.465
VO_2_ max/weight (mL/kg/min)	0.206	0.397	−0.055	0.822	−0.119	0.629
VO_2_ max % predicted	0.197	0.418	−0.098	0.689	−0.099	0.686
VO_2_-AT (mL/min)	0.265	0.273	0.500	0.029	0.209	0.391
VO_2_-AT/weight (mL/kg/min)	0.330	0.168	0.616	0.005	0.344	0.149
RER	0.014	0.956	−0.349	0.143	−0.156	0.523
VE/VCO_2_	−0.193	0.428	0.162	0.506	0.348	0.145
ΔVO_2_/ΔWR (mL/min/Watt)	0.060	0.808	−0.138	0.574	−0.143	0.559
WR (Watt)	0.016	0.948	−0.098	0.691	−0.151	0.537
WR % predicted	0.145	0.553	−0.063	0.799	−0.173	0.479
Predicted O_2_ pulse	−0.493	0.032	−0.261	0.281	−0.154	0.530
O_2_ pulse (mL/beat)	0.134	0.584	−0.129	0.598	−0.205	0.401
O_2_ pulse % predicted	0.293	0.223	−0.023	0.926	−0.112	0.647
HR max (bpm)	−0.077	0.754	−0.045	0.855	0.048	0.844
HR max % predicted	−0.065	0.792	−0.041	0.868	0.035	0.886
SBP max (mmHg)	0.026	0.915	0.159	0.515	0.146	0.551
DBP max (mmHg)	−0.178	0.466	−0.074	0.764	0.054	0.828

**Table 5:** Serum and salivary cortisol determinations (*p*-values were adjusted by Bonferroni correction).

	T0 Serum Cortisol (ng/mL)		T1 Serum Cortisol (ng/mL)	
	r	*p*	r	*p*
T0 salivary cortisol (µg/dL)	0.720	0.002	0.534	0.036
T2 salivary cortisol (µg/dL)	0.569	0.022	0.872	<0.001
T3 salivary cortisol (µg/dL)	0.303	0.414	0.765	<0.001

## Data Availability

Not applicable.
